# Parental Bipolar Symptoms and Identity Development in Emerging Adults: The Mediating Role of Parental Attachment

**DOI:** 10.3390/bs16040561

**Published:** 2026-04-09

**Authors:** Alexa D. Loonam, Casey Andrion, Steven L. Berman

**Affiliations:** Department of Psychology, University of Central Florida, Sanford, FL 32773, USA; alexa.loonam@ucf.edu (A.D.L.); cassandra.andrion@ucf.edu (C.A.)

**Keywords:** parental bipolar symptoms, identity development, attachment, parental psychopathology

## Abstract

Bipolar disorder is characterized by extreme mood fluctuations that may create emotionally inconsistent caregiving environments for children. Although children of caregivers with bipolar disorder are at elevated risk for psychosocial difficulties, less is known about how parental bipolar symptoms (PBSs) relate specifically to identity development. The present study investigated associations between perceived PBSs and identity outcomes among emerging adults, examining parental attachment as a potential mediator. College students (*N* = 399) completed an anonymous online survey assessing identity development, attachment to parents, and perception of PBSs. PBSs were positively associated with identity distress, disturbed identity, and lack of identity, and negatively associated with identity consolidation. Mediation analyses indicated that parental attachment partially or fully mediated the relationships between PBSs and each identity variable, suggesting that higher levels of PBSs were associated with less secure attachment, which in turn were linked to greater identity difficulties. These findings highlight the role of parental mental health and attachment in shaping identity development and underscore the importance of accessible mental health care for youth navigating identity formation in the context of caregiver psychopathology. Clinical implications and future directions are discussed.

## 1. Introduction

### 1.1. Background

Bipolar disorder, marked by extreme mood fluctuations, affects millions of adults and may expose children to inconsistent emotional environments (Nierenberg et al., 2023; Phillips & Kupfer, 2013; World Health Organization, 2024). While children of caregivers with bipolar disorder are at elevated risk for attachment difficulties and behavioral challenges (Lapalme et al., 1997; Madigan et al., 2006), less is known about how parental bipolar symptoms specifically relate to their child’s identity formation. Identity formation is a central developmental task during adolescence and emerging adulthood, during which individuals establish a coherent sense of self (Erikson, 1950, 1968). Because healthy identity development is strongly influenced by early attachment and adverse experiences (Armsden & Greenberg, 1987; Berman & Weems, 2016; Hatano et al., 2022), exposure to unpredictable caregiving (e.g., mood instability) may hinder this process and increase a child’s vulnerability to identity problems (Schwartz & Petrova, 2018). This study examines the relationship between parental bipolar symptoms and identity problems in their emerging adult children, exploring parental attachment as a potential mediator.

### 1.2. Identity

Identity is a multifaceted construct encompassing an individual’s roles, goals, values, and beliefs, shaping their sense of self and distinctiveness (Berman et al., 2020; Talaifar & Swann, 2018). Erikson’s Theory of Psychosocial Development posits that identity formation is a core developmental task during adolescence, involving the exploration and commitment to personal values and goals, offering coherence and direction in life (Erikson, 1950). Building on Erikson’s foundation, J. Marcia (1966) operationalized identity development into four distinct statuses: diffusion (low commitment and low exploration), foreclosure (high commitment and low exploration), moratorium (low commitment and high exploration), and achievement (high commitment and high exploration). These statuses are characterized by an individual’s level of exploration (actively seeking out and considering identity options) and commitment (making definitive choices about identity). In addition to these developmental processes, researchers have also examined identity distress, which refers to the subjective distress or discomfort individuals experience regarding difficulties resolving identity-related concerns (American Psychiatric Association, 1980; Berman & Weems, 2016).

Luyckx et al. (2008) refined this model by distinguishing between adaptive processes (exploration in breadth, exploration in depth, commitment-making, and identification with commitment) and maladaptive processes (ruminative exploration). Adaptive exploration reflects how well an individual explores identity options and commits to and truly identifies with the self-concept, whereas ruminative exploration reflects difficulties in the developmental process in which individuals repeatedly revisit identity questions without making progress toward commitment. To further expand upon traditional identity theories, Kaufman et al. (2015) operationalized identity development into three states: consolidated identity, disturbed identity, and lack of identity. A consolidated identity reflects a stable, coherent, and well-defined sense of self, and how well integrated one’s identity is to their self-concept. In contrast, a disturbed identity is marked by instability in self-concept, such as inconsistent goals, values, and relationships. Lack of identity describes a more severe absence of clarity and stability, in which individuals may feel disconnected or uncertain about who they are. Although related, these constructs capture different aspects of identity functioning as compared to identity distress. Whereas Kaufman et al.’s (2015) dimensions assess the structural organization and coherence of identity (e.g., consolidated, disturbed, or lack of identity), identity distress reflects the subjective emotional experience associated with difficulties integrating aspects of the self into a coherent sense of identity (American Psychiatric Association, 1980; Berman & Weems, 2016). Together, these constructs provide a complete understanding of identity formation because they consider both how identity is organized and how the individual feels about it.

Contemporary research emphasizes that identity is both stable and adaptive, evolving in response to life changes and social experiences (Schwartz et al., 2015). In this context, cognitive and social learning processes are integral to identity formation, as individuals continuously adapt their sense of self concerning their changing environment, social interactions, and life experiences (Oyserman et al., 2012). Thus, identity serves as a lens through which individuals interpret their past, navigate the present, and envision their future.

### 1.3. Bipolar Disorder

Bipolar disorder is a severe psychiatric condition classified as a mood disorder in the Diagnostic and Statistical Manual of Mental Disorders, Fifth Edition, Text Revision (American Psychiatric Association, 2022). It is characterized by the presence of distinct mood episodes, including manic, hypomanic, and depressive episodes. A diagnosis of bipolar I disorder requires at least one lifetime manic episode, defined by a period of abnormally elevated, expansive, or irritable mood and increased energy or activity lasting at least one week and accompanied by symptoms such as grandiosity, decreased need for sleep, pressured speech, distractibility, and increased goal-directed behavior. Bipolar II disorder involves at least one hypomanic episode and one major depressive episode, without a history of full mania. Major depressive episodes are characterized by persistent low mood or loss of interest, along with cognitive and physical symptoms such as fatigue, impaired concentration, and feelings of worthlessness. These mood disturbances can result in significant impairment in functioning (American Psychiatric Association, 2022; Goes, 2023; Goodwin & Jamison, 2007; Maassen et al., 2018; McElroy et al., 1996).

Children raised by caregivers exhibiting bipolar symptomatology may be particularly vulnerable to emotional unpredictability, inconsistent caregiving, and relational instability (Madigan et al., 2006). These fluctuations can undermine the stability of interpersonal relationships and disrupt the development of secure attachment, which is foundational to healthy psychological development (Bretherton, 1992). As the development of a stable identity is strongly influenced by early attachment experiences (Armsden & Greenberg, 1987), the emotional and relational instability associated with parental bipolar disorder may complicate this process in children.

### 1.4. Parental Psychopathology and Child Development

Children raised by caregivers with mental health disorders often face emotional, psychological, and social challenges that can disrupt healthy development. For instance, parental conditions such as obsessive–compulsive disorder, schizophrenia, borderline personality disorder (BPD), narcissistic personality disorder, anxiety, and depression have all been associated with increased risk for emotional dysregulation, insecure attachment, and mental health difficulties in children (Beidel et al., 2021; Bornovalova et al., 2018; Evans et al., 2015; Lebowitz et al., 2012). Notably, psychological features associated with these disorders are closely linked to problems in identity development in affected individuals themselves, particularly in disorders such as BPD and schizophrenia, where identity fragmentation and confusion are central features (American Psychiatric Association, 2022; Kernberg, 1970; Potterton et al., 2021). However, less is known about how parental psychopathology, particularly bipolar symptomatology, may impact identity development processes in their children.

### 1.5. The Relationship Between Adverse Experiences and Identity

Adverse experiences, particularly those involving family dysfunction or trauma, can significantly influence identity development by shaping how individuals perceive themselves and their social roles (Swann & Bosson, 2008). Integrating an identity is a dynamic process shaped by ongoing social interactions; therefore, models of severe emotional or relational dysfunction as a result of a caregiver’s bipolar symptomatology could have a substantial effect on their children’s self-perception, and, in turn, identity development depending on how these experiences are uniquely processed and integrated into their sense of self (Berman et al., 2020). An example of this is the phenomenon of *parentification*, in which children take on caregiving roles to meet the emotional needs of a mentally ill parent, often at the expense of their own developmental and emotional needs (Van Parys et al., 2014). This role reversal can interfere with emotional regulation and autonomy, contributing to identity confusion and distress, or even difficulties establishing relational boundaries in adulthood (Champion et al., 2009; Chase, 1999). Although research on the direct relationship between parental mental illness and child identity development is limited, existing evidence suggests that long-term exposure to caregiver stress and emotional unpredictability as a result of parental psychopathology may disrupt self-concept during this critical developmental stage and may also contribute to persistent psychological difficulties (Focht-Birkerts & Beardslee, 2000).

### 1.6. The Role of Parental Attachment

Family-based challenges, such as in cases of child abuse or neglect, can significantly disrupt identity development by damaging trust in the parent–child relationship, as well as complicating the formation of healthy relationships (Cicchetti & Toth, 2005), suggesting that similar adverse familial experiences resulting from parental psychopathology (e.g., emotional and relational instability) could have similar associations with identity. Contemporary research underscores that secure parental attachment is positively associated with adaptive identity development. In contrast, insecure or unresolved attachment patterns are linked to greater identity diffusion and confusion in adolescence and young adulthood (Gander et al., 2025). Furthermore, recent work connects parental attachment quality with adolescent identity status, such that supportive, communicative parental relationships are associated with coherent identity exploration and reduced foreclosure (Tizazu et al., 2026). Early attachment experiences also shape “internal working models” of attachment figures that influence self-concept and relational experiences across development (Bowlby, 1969; Armsden & Greenberg, 1987). Thus, secure attachment with caregivers, characterized by trust and open communication in the absence of alienation, supports psychological adjustment, clarity on self-concept, and even physical health (Greenberg et al., 1983). Positive and independent parent–child relationships also support high levels of ego-identity (J. E. Marcia, 1980), further supporting overall self-concept clarity. Therefore, modern literature and theoretical evidence suggest that parental attachment is crucial for healthy identity formation, and challenges that arise as a result of parental mental health difficulties, specifically those experienced by individuals diagnosed with bipolar disorder, may undermine the attachment relationship, which, in turn, may be associated with identity problems in their children.

### 1.7. The Present Study

The literature discussed above supports the idea that mental health challenges significantly impact individual and community well-being (Osborn et al., 2020). Despite established theoretical and relational links between parental mental illness and identity development (Van Parys et al., 2014), less is known about the specific effects of bipolar symptomatology, as bipolar disorder’s episodic nature presents unique challenges (Bornovalova et al., 2018). Most studies primarily focus on general psychological outcomes in the individual diagnosed or their children (e.g., attachment security, emotion regulation, or genetic predisposition to developing the disorder) rather than emerging adult identity development as a distinct construct.

Given identity formation’s reliance on stable attachment (Armsden & Greenberg, 1987), the present study examined how parental bipolar symptoms influence their child’s identity through the quality of the parent–child relationship. By understanding individuals’ unique challenges in forming a coherent sense of self amid caregiver mental instability, clinicians can better support identity development and emotional well-being during critical developmental phases.

The present study tested the following hypotheses: (H1) parental bipolar symptoms will be positively correlated with their emerging adult’s identity distress, disturbed identity, and lack of identity, while demonstrating a negative correlation with identity consolidation (Armsden & Greenberg, 1987; Berman et al., 2020; Chase, 1999; Kaufman et al., 2015; Madigan et al., 2006; Van Parys et al., 2014); (H2) parental bipolar symptoms will be negatively correlated with parental attachment (Bretherton, 1992; Cicchetti & Toth, 2005; Madigan et al., 2006); and (H3) parental attachment will function as a mediator in the association between parental bipolar symptoms and emerging adult identity problems, such that higher levels of parental bipolar symptoms will be associated with less secure parental attachment, which, in turn, will predict greater problems in identity formation (Armsden & Greenberg, 1987; Berman et al., 2020; Chase, 1999; Gander et al., 2025; Kaufman et al., 2015; Madigan et al., 2006; Tizazu et al., 2026; Van Parys et al., 2014).

## 2. Materials and Methods

### 2.1. Participants

Participants were 399 college students (*M* = 20.56, *SD* = 5.45) recruited from psychology courses at a large university in Florida, USA, through SONA, an online research participation platform. Eligibility for this study required university affiliation and a minimum age of 18 years. The sample was 68.2% female, 27.8% male, 2% non-binary, 1.5% transgender, and 0.5% identified as “other.” Ethnic and racial composition included 48.6% White, 24.8% Hispanic/Latinx, 10% Asian, 7% African American, 7.8% Multiracial, and 1.8% “other.” The class standing of the sample consisted of 46.1% freshmen, 21.3% sophomores, 22.1% juniors, 8.8% seniors, 0.5% non-degree-seeking students, 0.3% graduate students, and 1% who reported “other.” All procedures were approved by the university’s Institutional Review Board (IRB) and adhered to APA ethical guidelines.

### 2.2. Procedure

First, the project was submitted for approval to the university’s IRB. After receiving IRB approval, participants were recruited through SONA. Students can earn course credit by participating in research. They had the option to select from a list of available studies, including the current study. Those who selected this study were directed to an online survey battery in Qualtrics. At the start of the survey, participants were provided an Explanation of Research. The online survey ensured anonymity, with no personal identifiers collected. Those who chose not to participate were redirected to the end of the survey battery, where no data was gathered. Prior to analyses, incomplete responses were excluded from the sample. Multiple attention check items were embedded throughout the survey to assess response validity. Additionally, participants were asked at the conclusion of the survey whether their data should be used for research purposes. Responses from participants who failed attention checks or indicated that their data should not be used were removed from the final sample.

### 2.3. Measures

Participants completed a brief demographic questionnaire assessing gender identity, age, race/ethnicity, and class standing. They were also instructed to identify a single “primary caregiver” on whom to base responses for all parental measures, defined as the individual most responsible for their upbringing.

Parental bipolar symptoms were assessed using the General Behavior Inventory—Parent Version (GBI-P; Youngstrom et al., 2008). The GBI-P is a self-report questionnaire consisting of a series of items that respondents rate on a 4-point Likert-type scale (1 = *Never or Hardly Ever*, 2 = *Sometimes*, 3 = *Often*, 4 = *Very Often or Almost Constantly*) based on their experiences over an extended period. This is a psychological assessment tool designed to measure the presence and severity of mood disorders, specifically focusing on manic and depressive symptoms. Originally developed by Depue et al. (1989), it has been widely used in both clinical and research settings to identify bipolar spectrum disorders and unipolar depression. Using the latter adapted version by Youngstrom et al. (2008), this 10-item questionnaire was adapted for this study so that participants could answer questions regarding their “primary caregivers’” behaviors rather than their own. A sample item is: “*Has your primary caretaker had periods lasting several days or more when he/she felt depressed or irritable, and then other periods of several days or more when he/she felt extremely high, elated, and overflowing with energy?*” Youngstrom et al. (2008) reported a Cronbach’s alpha of 0.92 for this measure. In the present study, the internal reliability was 0.91.

Parental attachment was assessed using the parent subscale of the Inventory of Parent and Peer Attachment—Revised (IPPA-R; Armsden & Greenberg, 1987; Gullone & Robinson, 2005). The IPPA-R assesses trust, quality of communication, and feelings of anger or alienation with parents and peers using a 5-point Likert-type scale (1 = *Almost Never or Never True*, 2 = *Not Very Often True*, 3 = *Sometimes True*, 4 = *Often True*, 5 = *Almost Always or Always True*). Participants were asked to report their feelings about their “primary caregiver.” A sample item is: “*I don’t get much attention from my primary caretaker.*” Gullone and Robinson (2005) reported a Cronbach’s alpha of 0.85 for parental trust, 0.79 for parental communication, and 0.81 for parental alienation. Using the formula developed by the authors of the measure, a total score for healthy parental attachment was calculated by adding the scores for trust and communication and subtracting the alienation score, thus creating a single attachment score that reduces the number of statistical tests and thereby decreases the risk of Type I error. Previous research using the IPPA has similarly combined these dimensions into an overall index of attachment quality because the subscales show strong intercorrelations and together represent overall parental attachment security (Armsden & Greenberg, 1987; Pace et al., 2011). The internal reliability for the total score in this study was 0.84.

Identity distress was assessed using the Identity Distress Scale (IDS; Berman et al., 2004). The IDS is designed to measure distress and discomfort related to identity. Respondents rate their level of distress on a 5-point Likert-type scale (1 = *Not at All*, 2 = *Mildly*, 3 = *Moderately*, 4 = *Severely*, 5 = *Extremely*) across seven domains of identity, including long-term goals, career choice, friendships, sexual orientation and behavior, religion, values or beliefs, and group loyalties. Berman et al. (2004) reported an internal consistency of 0.84, with a test–retest reliability of 0.82. In the present study, Cronbach’s alpha was 0.82.

Identity stability was assessed using the Self-Concept and Identity Measure (SCIM; Kaufman et al., 2015). The SCIM is a 27-item scale comprising three subscales: identity consolidation, disturbed (unstable) identity, and lack of identity. Participants respond by rating their level of agreement with each statement using a 7-point Likert scale (1 = *Strongly Disagree*, 2 = *Disagree*, 3 = *Slightly Disagree*, 4 = *Neutral*, 5 = *Slightly Agree*, 6 = *Agree*, 7 = *Strongly Agree*). A sample item is: “*I try to act the same as the people I’m with (interests, music, dress) and I change that all the time.*” Bogaerts et al. (2018) reported internal consistency coefficients of 0.71, 0.85, and 0.92 for identity consolidation, disturbed identity, and lack of identity, respectively. Alpha coefficients for identity consolidation, disturbed identity, and lack of identity in the present study were 0.76, 0.88, and 0.80, respectively. Although these subscales are related, they capture conceptually distinct aspects of identity organization: consolidation reflects a coherent and well-integrated sense of self, disturbed identity reflects instability or inconsistency in self-concept, and lack of identity reflects a more severe absence of clarity and direction. Accordingly, the subscales were analyzed as separate observed variables to preserve the unique information captured by each dimension.

### 2.4. Data Analysis

For this cross-sectional correlational study, preliminary analyses (descriptive statistics, reliability, and correlations) and a regression-based mediation model were conducted using IBM SPSS Statistics Version 29.0.2.0 (20).

## 3. Results

### 3.1. Preliminary and Descriptive Analyses

Bivariate correlations among all study variables are reported in Table 1. Independent sample *t*-tests revealed that women reported higher levels of identity distress (*t* = −2.60, *p* = 0.01) and lack of identity (*t* = −2.36, *p* = 0.02) than men. No other gender differences were observed. A one-way analysis of variance (ANOVA) indicated significant racial/ethnic differences in disturbed identity (*F*_(5, 393)_ = 2.65, *p* = 0.02). Scheffé post hoc analyses revealed that Asian participants scored lower in disturbed identity than Hispanic/Latinx participants (*p* = 0.03). Class standing was also tested using a one-way ANOVA, which showed no significant differences. Age and measure relationships were analyzed using Pearson correlations, resulting in a statistically significant negative relationship for disturbed identity (*r* = −0.11, *p* = 0.03).

### 3.2. Main Analyses

First, Pearson product-moment bivariate correlational analyses were used to test whether parental bipolar symptoms would be positively correlated with emerging adult identity distress, disturbed identity, and lack of identity while demonstrating a negative correlation with identity consolidation. A significant positive correlation emerged between parental bipolar symptoms and identity distress (*r* = 0.29, *p* < 0.001), disturbed identity (*r* = 0.29, *p* < 0.001), and lack of identity (*r* = 0.24, *p* < 0.001), while demonstrating a negative correlation with identity consolidation (*r* = −0.23, *p* < 0.001). Thus, the hypothesis was supported, indicating that parental bipolar symptoms are significantly associated with identity difficulties (identity distress, disturbance, and lack) and hold a negative relationship with a stable sense of self (identity consolidation).

Second, a Pearson product-moment bivariate correlational analysis was used to test whether parental bipolar symptoms would be negatively correlated with parental attachment. Findings revealed a significant negative correlation between parental bipolar symptoms and parental attachment (*r* = −0.49, *p* < 0.001). Therefore, the hypothesis was supported, indicating that a negative relationship between the severity of parents’ bipolar disorder symptoms and the intensity of secure attachment to parents was confirmed.

**Figure 1:**
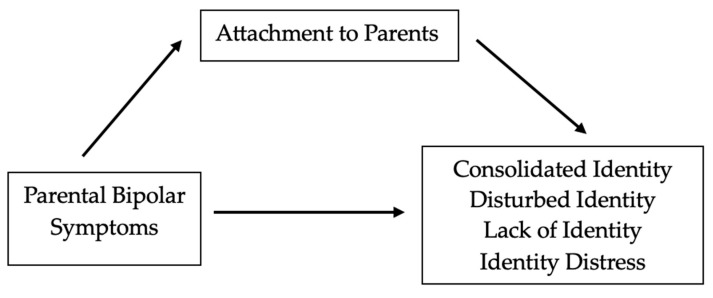
Regression-Based Mediation Model.

Third, a series of regression-based mediation analyses (Holmbeck, 1997) was used to test whether parental attachment would mediate the relationship between parental bipolar symptoms and the identity variables (see Figure 1). In all regressions, age and gender were entered on Step 1 as control variables. In the first regression equation, parental bipolar symptoms predicted attachment (*F*_(3, 379)_ = 40.10, *p* < 0.001). This fulfilled the first requirement of mediation. To meet the next requirement, four regression analyses were conducted with one of the identity variables serving as the dependent variable in each regression equation. In Step 2 of the hierarchical regressions, after controlling for age and gender identity, the parental bipolar symptom score was added, and in Step 3, the attachment score was added to the equation. To establish full mediation according to Holmbeck, parental bipolar symptoms should be a significant predictor in Step 2, but lose significance in Step 3, leaving only attachment as the significant predictor. If parental bipolar symptoms remain significant on Step 3, but greatly reduce in significance, this would be indicative of partial mediation. Step 3 predictor coefficients for each identity variable are shown in Table 2. For consolidated identity, parental bipolar symptoms were a significant predictor in Step 2 (*β* = −0.24, *t* = -4.89, *p* < 0.001) but were no longer significant on Step 3, suggesting full mediation. For disturbed identity, parental bipolar symptoms were a significant predictor in Step 2 (*β* = 0.29, *t* = 5.83, *p* < 0.001) and remained significant in Step 3 but less so (*β* = 0.16, *t* = 2.88, *p* = 0.004), suggesting partial mediation. For lack of identity, parental bipolar symptoms were a significant predictor in Step 2 (*β* = 0.25, *t* = 4.98, *p* < 0.001) but were no longer significant on Step 3, suggesting full mediation. For identity distress, parental bipolar symptoms were a significant predictor in Step 2 (*β* = 0.29, *t* = 6.00, *p* = 0.001) and remained significant in Step 3 but less so (*β* = 0.16, *t* = 3.06, *p* = 0.002), suggesting partial mediation.

## 4. Discussion

The present study explored the relationship between parental bipolar symptomatology and identity development, with a focus on parental attachment as a mediating mechanism. Preliminary analyses revealed some gender and racial/ethnic differences in identity variables. Specifically, women in this sample reported higher levels of identity distress and lack of identity than men, and Asian participants reported lower levels of disturbed identity compared with Hispanic/Latinx participants. These findings were exploratory and may reflect socio-cultural or contextual factors that influence identity development, such as differential socialization experiences, cultural norms around self-expression, or the salience of identity processes in specific groups, rather than inherent differences. Consistent with hypotheses, individuals who perceived their caregivers as exhibiting bipolar symptoms reported greater identity difficulties, including higher levels of identity distress, disturbed identity, and lack of identity, as well as lower levels of identity consolidation. These findings are consistent with prior work demonstrating that chronic emotional unpredictability, a key symptom of bipolar disorder (American Psychiatric Association, 2022), within the caregiving environment is associated with disruptions in identity-related processes, particularly maladaptive forms of exploration and difficulty achieving identity coherence (Berman et al., 2020; Luyckx et al., 2008; Madigan et al., 2006; Schwartz & Petrova, 2018).

Importantly, parental attachment mediated this relationship. Higher levels of parental bipolar symptoms were linked to less secure attachment, which, in turn, predicted greater identity distress, disturbed identity, and lack of identity, as well as lower consolidated identity. This finding aligns with attachment theory, which posits that early caregiving relationships lay the foundation for later self-concept and identity development (Armsden & Greenberg, 1987; Bowlby, 1969). More recent research similarly suggests that insecure attachment in the familial context is associated with identity instability, increased identity distress, and difficulty integrating values and goals during emerging adulthood (Cicchetti & Toth, 2005). Thus, the present findings build on attachment theory and extend prior attachment research by suggesting that parental bipolar symptomatology may represent a specific relational context of attachment insecurity and identity problems. While the mediation model is consistent with the possibility that parental bipolar symptoms are related to identity outcomes through parental attachment, the cross-sectional nature of the data precludes conclusions about causal direction. Alternative interpretations are also plausible; for instance, individuals experiencing identity problems may retrospectively perceive their parents as more emotionally unpredictable.

Although attachment partially mediated this relationship for some identity outcomes (e.g., disturbed and distress), these partial effects remain theoretically meaningful. It suggests that while disruptions in the attachment bond explain a portion of how parental bipolar symptoms affect identity, additional pathways are likely to exist. These may include broader family functioning variables (e.g., parental warmth, boundary-setting, communication patterns) or the emerging adult’s individual personality traits that influence resilience and self-definition. Prior research has shown that children of parents with chronic mental illness often experience role confusion or parentification, which may interfere with autonomy development and identity consolidation independent of attachment insecurity (Champion et al., 2009; Chase, 1999; Van Parys et al., 2014). Thus, these findings point to a possible multifactorial process through which parental psychopathology may contribute to identity outcomes in their children.

These results reinforce the broader developmental implications of being raised in environments amid parental mental illness. Beyond immediate emotional or behavioral outcomes, such experiences appear to shape how individuals understand themselves. Consistent with contemporary models of identity as a dynamic and context-sensitive process (Oyserman et al., 2012; Schwartz et al., 2015), the present study suggests that ongoing exposure to caregiver mood instability may exert long-term effects on self-concept development during critical periods of identity formation, such as late adolescence and emerging adulthood (Arnett, 2014; Erikson, 1968). By understanding identity problems as a meaningful outcome associated with parental bipolar symptoms, these findings extend existing research that has largely focused on emotional and behavioral adjustment, highlighting the development of a healthy self-concept as an important domain for future research and clinical intervention.

### 4.1. Limitations and Future Research

While this study advances our understanding of how parental bipolar symptomatology relates to identity development, several limitations must be acknowledged. First, the exclusive use of self-report measures introduces the potential for recall bias, particularly in retrospective evaluations of parental behavior. Additionally, the sample consisted solely of college students, and while this is a prime age and context for identity development, the findings may not generalize to broader, more diverse populations. Future studies would benefit from larger, more representative samples to enhance external validity. Another limitation lies in the unmonitored nature of the online data collection process, which could have affected the accuracy and consistency of responses. Employing structured, in-person assessments or interviews in future research could help ensure greater engagement and data reliability.

Furthermore, the study relied on participants’ self-reported perceptions of parental bipolar symptoms rather than confirmed clinical diagnoses or parental self-reports. As a result, the measure may reflect participants’ perceptions of parental emotional instability rather than clinically verified bipolar symptomatology, and it limits the ability to examine symptom severity or diagnostic nuance. Future research should aim to include participants with verified parental diagnoses and consider symptom-level variation and caregiver type (e.g., mother, father, grandparent, adopted parent) to clarify these effects. Finally, the study did not assess developmental differences in identity formation, and the cross-sectional design precludes conclusions about directionality or causality. Longitudinal research could offer critical insight into how identity challenges emerge and evolve across adolescence and emerging adulthood in the context of parental mental illness. By addressing these limitations, future investigations can build on the present findings to more fully capture the complexity of identity development in the face of familial mental health challenges.

### 4.2. Implications for Practice

Despite limitations in study design, these results still carry important clinical implications given the prevalence of bipolar disorder among adults (Nierenberg et al., 2023; World Health Organization, 2024). Mental health professionals working with individuals raised in these caregiving environments should consider identity-related challenges and attachment disruptions as critical areas of concern. While interventions that strengthen emotional coping skills remain valuable, they should be complemented with approaches that promote relational stability (e.g., family intervention) and self-concept clarity.

Moreover, these findings underscore the need to raise awareness about how parental mental health disorders can disrupt key developmental processes in children, particularly identity formation. This research highlights the need for a more comprehensive understanding of how environmental instability affects not only immediate cognitive and behavioral processes but also long-term identity development. Clinicians may benefit from integrating attachment-informed frameworks and explicit identity-focused work (e.g., exploration of values, roles, and self-concept coherence) into treatment planning. Additionally, advocating for preventative measures that address the broader developmental impact of living with a parent with bipolar disorder or other mental illnesses is essential for the well-being of both the individual diagnosed and those with whom they share relationships.

## 5. Conclusions

The present study demonstrates that perceived parental bipolar symptoms are associated with increased identity problems and decreased identity consolidation in emerging adults, with parental attachment partially and fully mediating this relationship. These findings highlight the importance of considering both relational context and parental mental health when examining identity development. Clinically, supporting secure parent–child attachment and fostering self-concept clarity may mitigate identity-related challenges for individuals exposed to caregiver mood instability. Future research using longitudinal designs and verified clinical diagnoses is needed to further clarify these pathways and inform targeted interventions.

## Figures and Tables

**Table 1:** Pearson Bivariate Correlation Matrix of All Study Variables.

Study Variables	1	2	3	4	5
Parental Bipolar Symptoms	-				
2. Attachment to Parents	−0.49 *	-			
3. Identity Distress	0.29 *	−0.34 *	-		
4. Identity Consolidation	−0.23 *	0.41 *	−0.35 *	-	
5. Disturbed Identity	0.29 *	−0.34 *	0.45 *	−0.43 *	-
6. Lack of Identity	0.24 *	−0.41 *	0.48 *	−0.42 *	−0.64 *

*Note*: * *p* < 0.001.

**Table 2:** Step three regression coefficients predicting identity variables.

Variables	Consolidated Identity			Disturbed Identity			Lack of Identity			Identity Distress		
	*F*_(4, 378)_ = 19.90, *p* < 0.001			*F*_(4, 378)_ = 16.45, *p* < 0.001			*F*_(4, 378)_ = 20.87, *p* < 0.001			*F*_(4, 378)_ = 17.82, *p* < 0.001		
	***β***	***t***	***p***	***β***	***t***	***p***	***β***	***t***	***p***	***β***	***t***	***p***
Age	0.05	1.02	0.307	−0.12	−2.58	**0.010**	0.06	1.30	0.195	−0.09	−1.78	0.075
Sex	0.02	0.41	0.682	0.04	0.77	0.441	0.12	2.48	**0.014**	0.13	2.70	**0.007**
Parental Bipolar Symptoms	−0.06	−1.05	0.296	0.16	2.88	**0.004**	0.07	1.31	0.19	0.17	3.06	**0.002**
Attachment	0.39	7.22	**<0.001**	−0.27	−4.88	**<0.001**	−0.36	−6.76	**<0.001**	−0.26	−4.80	**<0.001**

*Note*: Significant *p* values are bolded.

## Data Availability

The data presented in this study are available upon request from the corresponding author.
